# Covid-19 infection and vaccination during first trimester and risk of congenital anomalies: Nordic registry based study

**DOI:** 10.1136/bmj-2024-079364

**Published:** 2024-07-17

**Authors:** Maria C Magnus, Jonas Söderling, Anne K Örtqvist, Anne-Marie Nybo Andersen, Olof Stephansson, Siri E Håberg, Stine Kjaer Urhoj

**Affiliations:** 1 Centre for Fertility and Health, Norwegian Institute of Public Health, Oslo, Norway; 2 Clinical Epidemiology Division, Department of Medicine, Solna, Karolinska Institutet, Stockholm, Sweden; 3 Department of Obstetrics and Gynaecology, Visby County Hospital, Visby, Sweden; 4 Department of Public Health, University of Copenhagen, Copenhagen, Denmark; 5 Department of Women's Health, Karolinska University Hospital, Solna, Stockholm, Sweden; 6 Department of Global Public Health and Primary Care, University of Bergen, Bergen, Norway; 7 Statistics Denmark, Copenhagen, Denmark

## Abstract

**Objectives:**

To evaluate the risk of major congenital anomalies according to infection with or vaccination against covid-19 during the first trimester of pregnancy.

**Design:**

Prospective Nordic registry based study.

**Setting:**

Sweden, Denmark, and Norway.

**Participants:**

343 066 liveborn singleton infants in Sweden, Denmark, and Norway, with an estimated start of pregnancy between 1 March 2020 and 14 February 2022, identified using national health registries.

**Main outcome measure:**

Major congenital anomalies were categorised using EUROCAT (European Surveillance of Congenital Anomalies) definitions. The risk after covid-19 infection or vaccination during the first trimester was assessed by logistic regression, adjusting for maternal age, parity, education, income, country of origin, smoking, body mass index, chronic conditions, and estimated date of start of pregnancy.

**Results:**

17 704 (5.2%) infants had a major congenital anomaly. When evaluating risk associated with covid-19 infection during the first trimester, the adjusted odds ratio ranged from 0.84 (95% confidence interval 0.51 to 1.40) for eye anomalies to 1.12 (0.68 to 1.84) for oro-facial clefts. Similarly, the risk associated with covid-19 vaccination during the first trimester ranged from 0.84 (0.31 to 2.31) for nervous system anomalies to 1.69 (0.76 to 3.78) for abdominal wall defects. Estimates for 10 of 11 subgroups of anomalies were less than 1.04, indicating no notable increased risk.

**Conclusions:**

Covid-19 infection and vaccination during the first trimester of pregnancy were not associated with risk of congenital anomalies.

## Introduction

Women infected with covid-19 during pregnancy have a higher risk of pregnancy complications.^1^ ^2^ ^3^ Based on this evidence, and studies showing that pregnant women are at increased risk of severe disease from covid-19,^3^ ^4^ the authorities in most countries recommend that pregnant women get vaccinated against covid-19.^5^ ^6^ ^7^ ^8^ Because pregnant women are not often included in randomised controlled trials of vaccines before marketing, evidence relating to the safety of vaccines during pregnancy relies on observational data after the introduction of vaccines. Vaccination of pregnant women against covid-19 was therefore recommended before conclusive safety data were available. Studies evaluating the safety of covid-19 vaccines among pregnant women after marketing have been reassuring, providing no evidence of an increased risk of pregnancy complications.^9^ ^10^ ^11^ ^12^

Limited evidence is available about the risk of major congenital anomalies after infection with^13^ ^14^ or vaccination against^15^ ^16^ ^17^ ^18^ ^19^ covid-19. A study of 92 pregnancies in women infected with covid-19 during the first trimester and 292 without covid-19 infection (data from the International Registry of Coronavirus Exposure in Pregnancy) indicated no increased risk of any major congenital anomalies (relative risk 1.2, 95% confidence interval (CI) 0.3 to 4.2).^13^ A registry based study from Scotland reported similar findings of no increased risk of congenital anomalies after infection with covid-19 (1574 with infection and 4722 without infection; adjusted odds ratio 0.94; 95% CI 0.57 to 1.54), in addition to no increased risk with covid-19 vaccination (6731 with vaccination and 20 193 without vaccination; adjusted odds ratio 1.00, 95% CI 0.81 to 1.22).^18^ An Israeli study of 24 288 pregnancies (2134 in women vaccinated against covid-19 during the first trimester) also reported no increased risk of congenital anomalies after vaccination (relative risk 0.69, 95% CI 0.44 to 1.04).^16^ A smaller study in the United States found that 27 of 534 infants whose mothers were unvaccinated against covid-19 and 109 of 2622 infants whose mothers were vaccinated any time during pregnancy were diagnosed with congenital anomalies (P value 0.35).^17^ Finally, a study of 1450 pregnancies in the COVI-PREG registry from Switzerland and France did not find evidence of an increased risk of congenital anomalies after vaccination against covid-19 during the first trimester (adjusted relative risk 0.89, 95% CI 0.12 to 6.80).^19^

Most of these studies had an inadequate sample size to robustly examine these rare outcomes,^13^ ^15^ ^17^ ^19^ were not able to study first trimester exposure,^15^ ^18^ or did not investigate subgroups of congenital anomalies.^15^ ^16^ ^17^ ^19^ For congenital anomalies, first trimester exposure is of particular interest.^20^ Because not all major congenital anomalies are detected at birth, follow-up information from the first year of life is important to reduce misclassification.^21^ As a consequence, it has only recently become possible to study these outcomes after covid-19 infection and vaccination in early pregnancy. The objective of this study was to study the risk of major congenital anomalies according to infection with or vaccination against covid-19 during the first trimester.

## Methods

### Study population

We studied liveborn singleton infants in Sweden, Denmark, and Norway with estimated start of pregnancy between 1 March 2020 and 14 February 2022. Births were identified through the Swedish Pregnancy Register,^22^ the Danish National Patient Register (registrations of international classification of disease, 10th revision (ICD-10) codes Z38, O80-84, and P95),^23^ and the Medical Birth Registry of Norway.^24^ The Danish and Norwegian data included all births nationally, while the Swedish data included 94% of all births (in 18 of 21 Swedish regions). We required a minimum of nine months (275 days) of postnatal follow-up (end of follow-up in the three different national registry linkages was 31 March 2023 for Sweden, 31 December 2022 for Denmark, and 15 September 2023 for Norway). To avoid oversampling of preterm pregnancies, we excluded births that were not able to reach 42 completed gestational weeks and have nine months of postnatal follow-up by the end of follow-up in the national linkages. We obtained information on maternal socioeconomic measures, infections with covid-19, and vaccination against covid-19 from national databases (see supplementary appendix for details).

### Covid-19 infection and vaccination

The exposures of interest were infection with or vaccination against covid-19 during the first trimester (13 weeks plus six days). The two exposures were evaluated separately. We did not evaluate the role of a combined exposure, or exclude those exposed to the other exposure of interest from the reference group. The start of pregnancy was estimated from the date of birth minus the gestational age in days (the gestational age of the pregnancy was estimated by ultrasound for more than 90% of births, or by day of last menstrual period). Information on laboratory confirmed polymerase chain reaction (PCR) positive tests for covid-19 was obtained from mandatory reports to SmiNet at the Public Health Agency for Sweden,^25^ from the Norwegian Surveillance System for Communicable Diseases for Norway,^26^ and information on PCR and antigen positive tests was obtained from the Microbiology Database at the State Serum Institute for Denmark.^27^ ^28^ In Denmark, positive antigen tests that were followed by a negative PCR test within four days were excluded, and 15% of the included positive tests were only based on antigen tests. Until around March to April 2022, pregnant women who tested positive on a self-administered antigen test were advised to obtain a confirmatory PCR test.

Information on vaccination was obtained from mandatory national vaccination registries. We restricted the analysis to the two mRNA vaccines from Pfizer-BioNTech (BNT162b2) and Moderna (mRNA-1273), and excluded women who had received other covid-19 vaccines. At the beginning of the covid-19 pandemic, vaccination during the first trimester was not recommended for the general population of pregnant women, but could be considered for those at high risk. Pregnant women were advised to get vaccinated from the second trimester onwards, starting from May 2021 in Sweden, July 2021 in Denmark, and August 2021 in Norway. Women vaccinated during the first trimester at the beginning of the pandemic include those who were vaccinated before realising they were pregnant, and those who had a particularly high risk of infection or severe disease from covid-19 owing to underlying chronic conditions or their job (ie, healthcare workers). General recommendations for pregnant women to get vaccinated during the first trimester started in January 2022 in Norway, but these recommendations were not issued in Sweden and Denmark. eTable 1 gives a brief overview of major changes to recommendations.

### Congenital anomalies

We defined major congenital anomalies identified during the first nine months of life according to the EUROCAT (European Surveillance of Congenital Anomalies) classification, guide 1.5.^29^ Information on congenital anomalies was based on data from the National Birth Registry (Norway), the Pregnancy Register (Sweden), and national patient registries (all countries). The national patient registries include all inpatient and outpatient contact with specialist healthcare services based on mandatory reporting. Four digit ICD-10 codes (QXX.XX) were not available in Norway and Sweden, and so three digit codes were used (QXX.X). Anomalies were categorised as any major congenital anomaly, congenital heart defects, nervous system anomalies, eye anomalies, ear, face and neck anomalies, respiratory anomalies, oro-facial clefts, gastrointestinal anomalies, abdominal wall defects, congenital anomalies of kidney and urinary tract, genital anomalies, and limb anomalies. eTable 2 presents the ICD-10 codes used to define the subgroups of anomalies. We do not show results when less than five infants with maternal exposure were reported across the three countries.

### Covariates

We obtained information on maternal age (<25, 25-29, 30-34, 35-39, and ≥40 years), parity (0, 1, ≥2), maternal educational level (≤9 years, 10-12 years, >12 years), household income based on the national distributions (in thirds), maternal region of birth (Scandinavia, other European countries, Middle East or Africa, other or unknown), estimated date of start of pregnancy (estimated as date of birth minus gestational age in days; continuous), smoking in pregnancy (yes or no), body mass index before pregnancy or early in pregnancy (World Health Organization categories: underweight, normal weight, overweight, obese, unknown), and pre-existing chronic condition before pregnancy (yes or no; included hypertension, chronic kidney disease, asthma, cardiovascular disease, thrombosis, diabetes, and epilepsy). eTable 3 presents information on the ICD-10 codes used to define these chronic conditions. All of these covariates were considered potential confounders for infection with and vaccination against covid-19.

### Statistical analysis

All analyses were conducted separately for each country, and subsequently combined using a random effects meta-analysis. We developed a detailed analysis plan harmonising the definition of all variables before starting the analysis, which was followed by the analysts in all three countries. Common analysis scripts were developed for Denmark and Norway using Stata, while separate scripts were developed for Sweden using SAS. Heterogeneity between the countries was examined using the I^2^ statistic. We do not show country specific estimates for legal reasons relating to small numbers of certain outcomes. We first examined the risk of congenital anomalies after infection with covid-19 during the first trimester using logistic regression. The multivariable model adjusted for maternal age at the start of pregnancy, parity, educational level, household income level, maternal region of birth, estimated date of start of pregnancy, smoking during pregnancy, body mass index before or early in pregnancy, any history of chronic conditions, and vaccination against covid-19 during the first trimester. We accounted for the dependency between multiple pregnancies to the same woman by using cluster variance estimation. We also explored differences in the risk of congenital anomalies according to the covid-19 viral variants using the same calendar time cut-off points for major circulating variants, as previously published.^30^

We also evaluated the risk of congenital anomalies according to vaccination against covid-19 during the first trimester. This analysis was restricted to pregnancies starting after 1 January 2021 when vaccines became available. The reference group comprised women not vaccinated in the first trimester (including women vaccinated before pregnancy and after the first trimester). The multivariable model was adjusted for the same covariates as in analyses of covid-19 infection, in addition to infection with covid-19 during the first trimester. We also evaluated differences according to the two different mRNA vaccines (BNT162b2 and mRNA-1273), and conducted a sensitivity analysis that excluded infants of mothers who remained unvaccinated at the end of follow-up (21%).

For both exposures, we conducted two sensitivity analyses. In the first sensitivity analysis, we required at least 12 months of follow-up after birth.^21^ In the second sensitivity analysis, we excluded infants with congenital anomalies that are known to have a main genetic cause (defined by the following codes: Q619, Q751, Q754, Q771, Q772, Q780, Q796, Q821, Q85, Q87, Q90-99).^31^ The analyses were conducted using Stata version 17 (Statacorp, TX, USA) and SAS version 9.4 (SAS Institute, NC, USA).

### Patient and public involvement

Because this study was based on deidentified data from national health registries, it was not permitted or possible to contact any registered individuals directly. The advisory group responsible for national guidelines for vaccination of pregnant women against covid-19 at the Norwegian Institute of Public Health provided important feedback and was informed of preliminary findings throughout the project. The researchers did not have the necessary infrastructure or funding available to further pursue additional patient or public partnership for this specific project.

## Results

**Fig 1:**
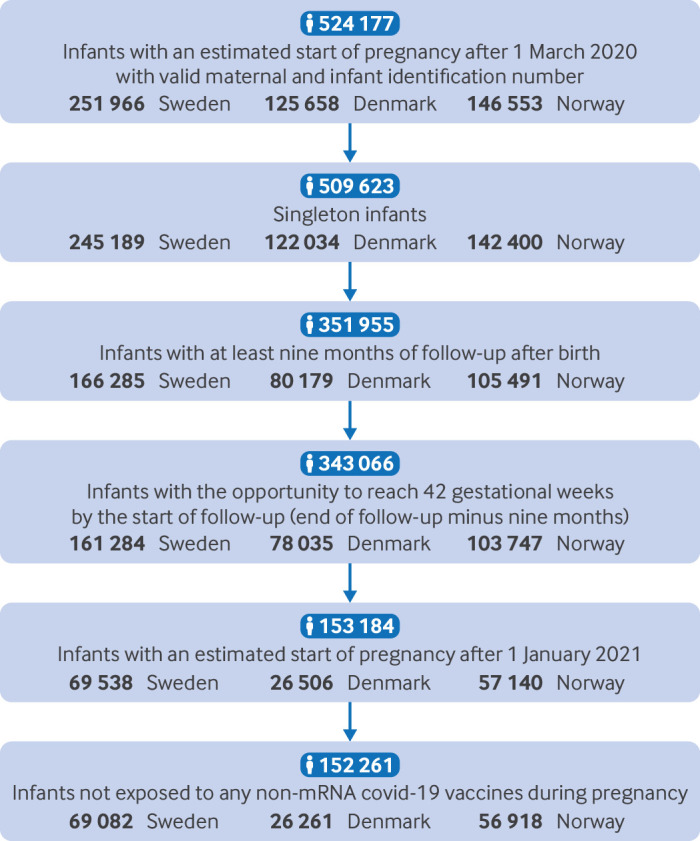
Study population

**Fig 2:**
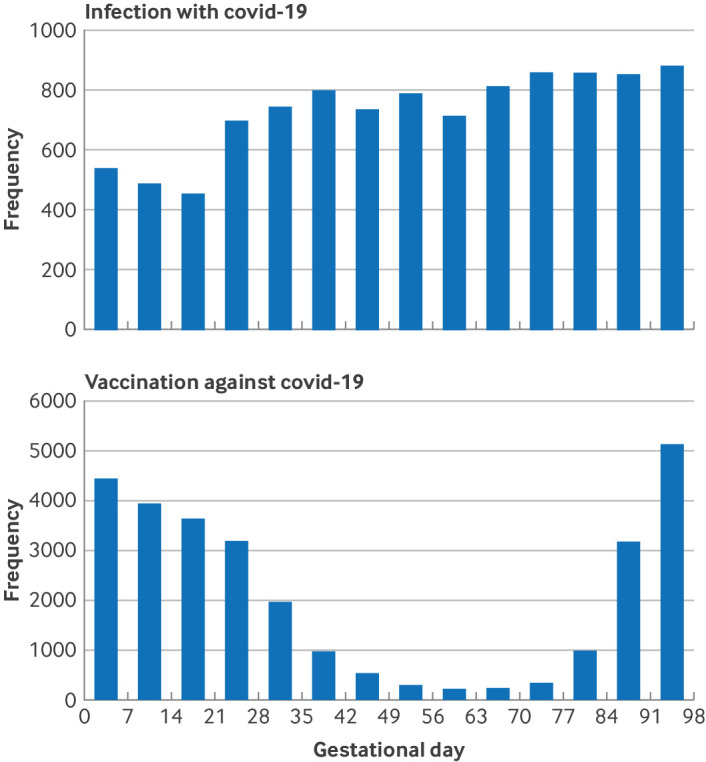
Gestational age distribution of infection with and vaccination against covid-19 during first trimester

A total of 343 066 singleton infants were included in the analysis of covid-19 infection (fig 1). The distribution of background characteristics was very similar across the three countries (table 1). There was a slightly higher proportion of infants born to women from the Middle East or Africa in Sweden, while the proportion of mothers with an underlying chronic disease appeared to be lower in Denmark than in Sweden and Norway. A total of 17 704 infants were diagnosed with a major congenital anomaly within a nine month follow-up, corresponding to 516 per 10 000 live births. Only 737 out of 17 704 (4.2%) had anomalies in two or more major congenital anomaly subgroups. Table 2 shows the rates of major congenital anomalies. Figure 2 shows the gestational age distributions for date of covid-19 infection (date of registered positive test) and vaccination against covid-19 during the first trimester, while eFigure 1 shows the distribution of calendar time for the start of pregnancy, infection with and vaccination against covid-19 during the first trimester.

**Table 1:** Maternal background characteristics among infants in each country

Maternal background characteristics	Sweden (n=161 284)	Denmark (n=78 035)	Norway (n=103 747)
**Age at start of pregnancy (years)**			
<25	17 292 (10.7)	7941 (10.2)	7863 (7.6)
25-29	53 082 (32.9)	28 830 (36.9)	31 117 (30.0)
30-34	59 915 (37.1)	27 787 (35.6)	41 357 (39.9)
35-39	25 749 (16.0)	11 207 (14.4)	19 227 (18.5)
≥40 years	5246 (3.3)	2270 (2.9)	4183 (4.0)
**Parity**			
0	68 641 (42.6)	36 641 (47.0)	44 361 (42.8)
1	60 431 (37.5)	29 563 (37.9)	38 709 (37.3)
2	22 094 (13.7)	9351 (12.0)	14 952 (14.4)
≥3	10 118 (6.3)	2480 (3.2)	5725 (5.5)
**Highest obtained educational level**			
≤9 years	9566 (5.9)	7877 (10.1)	13 793 (13.3)
10-12 years	50 524 (31.3)	20 661 (26.5)	19 786 (19.1)
>12 years	100 194 (62.1)	49 079 (62.9)	58 751 (56.6)
Unknown	1000 (0.6)	418 (0.5)	11 417 (11.0)
**Income category (thirds)**			
1st	52 532 (32.6)	20 779 (26.6)	32 296 (31.1)
2nd	52 533 (32.6)	24 294 (31.1)	32 263 (31.3)
3rd	52 533 (32.6)	25 478 (32.7)	32 083 (30.9)
Unknown	3686 (2.3)	7484 (9.6)	7105 (6.9)
Country of birth or origin*			
Scandinavia	115 061 (71.3)	65 308 (83.7)	76 839 (74.3)
Other European countries	12 485 (7.7)	5907 (7.6)	11 483 (11.1)
Middle East or Africa	26 113 (16.2)	3962 (5.1)	7333 (7.1)
Other or unknown	7625 (4.7)	2858 (3.7)	7776 (7.5)
**Any chronic disease**†			
No	139 864 (86.7)	75 266 (96.5)	93 681 (90.3)
Yes	21 420 (13.3)	2769 (3.6)	10 066 (9.7)
**Smoking during pregnancy**			
No	150 395 (93.2)	69 702 (89.3)	89 211 (86.0)
Yes	5024 (3.1)	5314 (6.8)	1734 (1.7)
Unknown	5865 (3.6)	3019 (3.9)	12 802 (12.3)
**Body mass index before pregnancy**			
<18.5	3351 (2.1)	2661 (3.4)	3196 (3.1)
18.5-24.9	81 545 (50.6)	41 512 (53.2)	56 307 (54.3)
25.0-29-9	43 842 (27.2)	19 893 (25.5)	23 652 (22.8)
≥30	26 561 (16.5)	12 542 (16.1)	14 633 (14.1)
Unknown	5985 (3.7)	1427 (1.8)	5959 (5.4)

* The other category includes North America, South America, Latin America, Asia, Australia, and New Zealand.

† Includes hypertension, chronic kidney disease, cardiovascular disease, asthma, thrombosis, diabetes mellitus (type 1 and 2), and epilepsy.

**Table 2:** Rates of EUROCAT (European Surveillance of Congenital Anomalies) categories of major congenital anomalies

EUROCAT categories of major congenital anomalies	Sweden	Denmark	Norway	Total or combined
Any	6963 (432)	4925 (631)	5816 (561)	17 704 (516)
Congenital heart defects	2133 (132)	922 (118)	1813 (175)	4868 (142)
Nervous system	186 (12)	129 (17)	128 (12)	443 (13)
Eye	337 (21)	91 (12)	188 (18)	616 (18)
Ear, face, and neck	68 (4)	33 (4)	44 (4)	145(4)
Respiratory	60 (4)	72 (9)	50 (5)	182 (5)
Oro-facial clefts	224 (14)	102 (13)	172 (17)	498 (15)
Gastrointestinal	570 (35)	333 (43)	288 (28)	1191 (35)
Abdominal wall defects	39 (2)	19 (2)	43 (4)	101 (3)
Kidney and urinary (CAKUT)	596 (37)	389 (50)	644 (62)	1629 (47)
Genital	673 (42)	321 (41)	484 (47)	1478 (43)
Limb	1087 (67)	1791 (230)	1084 (104)	3962 (115)

Data are numbers (rates per 10 000).

### Risk of congenital anomalies according to infection with covid-19

A total of 10 229 infants (3%) had mothers with covid-19 infection during the first trimester. These mothers had higher parity, lower educational level, lower household income level, and were more likely to be born in the Middle East or Africa (eTable 4). We did not find an increased risk of any major congenital anomalies after infection with covid-19 during the first trimester, with an adjusted odds ratio of 0.96 (95% confidence interval 0.87 to 1.05; table 3). Likewise, we did not find an increased risk of specific subgroups of congenital anomalies after maternal infection, with adjusted odds ratio ranging from 0.84 (0.51 to 1.40) for eye anomalies to 1.12 (0.68 to 1.84) for oro-facial clefts (table 3). There was some heterogeneity in the risk estimates for different congenital anomalies according to exposure to infection during the first trimester (I^2^ statistic: 11% for kidney and urinary anomalies, 29% for gastrointestinal anomalies, 50% for limb anomalies, and 0% for the other outcomes evaluated). Exposure to the index variant of covid-19 during the first trimester occurred in 3124 pregnancies, with 2065 to alpha and 5040 to delta variants. We observed no notable differences in the risk according to these three viral variants, although the results were associated with a high degree of uncertainty (eTable 5). Analyses among infants with at least 12 months of follow-up (compared with at least nine months in the main analysis), or excluding infants with presumed genetically related anomalies showed similar results (eTables 6 and 7).

**Table 3:** Risk of congenital anomalies according to infection with covid-19 during the first trimester

EUROCAT categories of major congenital anomalies*	Without maternal infection (n=332 837)	With maternal infection (n=10 229)	Odds ratio adjusted for estimated start of pregnancy (95% CI)	Fully adjusted odds ratio (95% CI)†
Any	17210	494	0.94 (0.86 to 1.04)	0.96 (0.87 to 1.05)
Congenital heart defects	4707	161	1.08 (0.92 to 1.27)	1.08 (0.92 to 1.28)
Nervous system	435	8	0.65 (0.32 to 1.30)	0.68 (0.33 to 1.37)
Eye	600	16	0.86 (0.52 to 1.42)	0.84 (0.51 to 1.40)
Oro-facial clefts	481	17	1.08 (0.66 to 1.77)	1.12 (0.68 to 1.84)
Gastrointestinal	1164	27	0.88 (0.50 to 1.55)	0.92 (0.55 to 1.54)
Kidney and urinary	1585	44	0.94 (0.70 to 1.27)	0.96 (0.69 to 1.33)
Genital	1436	42	0.92 (0.67 to 1.26)	0.94 (0.68 to 1.29)
Limb	3859	103	0.93 (0.71 to 1.21)	0.94 (0.69 to 1.27)

CI=confidence interval; EUROCAT=European Surveillance of Congenital Anomalies.

* Ear, face, and neck anomalies, respiratory anomalies, and abdominal wall defects were not evaluated because less than five infants with maternal exposure were reported across the three countries.

† Adjusted for maternal age, parity, highest obtained educational level, household income level, smoking during pregnancy, body mass index before pregnancy, country of birth or origin, estimated start of pregnancy (last menstrual period), chronic diseases, and vaccination against covid-19 in first trimester.

### Risk of congenital anomalies according to vaccination against covid-19

We included 152 261 infants in the analysis of congenital anomalies according to vaccination (fig 1). Among these, 29 135 (19%) had maternal exposure to covid-19 vaccination during the first trimester: 22 322 (77%) mothers received the BNT162b2 vaccine and 6813 (23%) had the mRNA-1273 vaccine. Mothers vaccinated against covid-19 during the first trimester had higher education and household income, were more likely to have an underlying chronic disease, and were more likely to be overweight or obese (eTable 8).

We did not find an increased risk of any major congenital anomaly among infants whose mothers were vaccinated against covid-19 during the first trimester, with an adjusted odds ratio of 1.03 (95% CI 0.97 to 1.09; table 4). When we examined subgroups of anomalies, adjusted estimates ranged from 0.84 (0.31 to 2.31) for nervous system anomalies to 1.69 (0.76 to 3.78) for abdominal wall defects (table 4). The estimates for 10 of 11 of the subgroups of anomalies were less than 1.04, indicating no notable increased risk. There was some heterogeneity in the estimates for the risk of the different congenital anomalies according to exposure to vaccination during the first trimester (I^2^ statistic: 32% for congenital heart defects, 40% for gastrointestinal anomalies, 44% for kidney and urinary anomalies, 76% for nervous system anomalies, and 0% for the other outcomes evaluated).

**Table 4:** Risk of congenital anomalies according to vaccination against covid-19 during the first trimester

EUROCAT categories of major congenital anomalies	Without maternal vaccination (n=123 126)	With maternal vaccination (n=29 135)	Odds ratio adjusted for estimated start of pregnancy (95% CI)	Fully adjusted odds ratio (95% CI)*
Any	6284	1395	1.01 (0.95 to 1.08)	1.03 (0.97 to 1.09)
Congenital heart defects	1745	416	1.02 (0.89 to 1.18)	1.04 (0.90 to 1.21)
Nervous system	147	30	0.80 (0.28 to 2.30)	0.84 (0.31 to 2.31)
Eye	227	53	0.88 (0.64 to 1.20)	0.87 (0.63 to 1.21)
Ear, face, and neck	57	6	0.44 (0.19 to 1.03)	0.44 (0.18 to 1.05)
Respiratory	72	9	0.68 (0.34 to 1.37)	0.67 (0.33 to 1.36)
Oro-facial clefts	179	46	1.12 (0.80 to 1.58)	1.03 (0.73 to 1.46)
Gastrointestinal	397	84	1.03 (0.74 to 1.44)	1.04 (0.74 to 1.46)
Abdominal wall defects	34	9	1.52 (0.69 to 3.33)	1.69 (0.76 to 3.78)
Kidney and urinary	593	134	1.03 (0.78 to 1.36)	1.01 (0.75 to 1.66)
Genital	322	72	0.95 (0.77 to 1.16)	1.00 (0.81 to 1.23)
Limb	1115	207	1.01 (0.88 to 1.16)	0.99 (0.86 to 1.14)

CI=confidence interval; EUROCAT=European Surveillance of Congenital Anomalies.

* Adjusted for maternal age, parity, highest obtained educational level, household income level, smoking during pregnancy, body mass index before pregnancy, country of birth or origin, estimated start of pregnancy (last menstrual period), chronic diseases, and infection with covid-19 in first trimester.

Restricting our analysis to infants with at least 12 months of follow-up, excluding infants with genetic disorders, or excluding infants of mothers who remained unvaccinated at the end of follow-up yielded similar results (eTables 9-11). We found no clear evidence of a difference in the risk of congenital anomalies according to exposure to the two different mRNA vaccines (eTable 12). Only 386 infants were exposed to infection and vaccination during the first trimester, therefore we did not evaluate the role of a combined exposure, or exclude those exposed to the other exposure of interest from the reference group.

### Including fetal deaths and late induced abortions

In Norway, we had information on all fetal deaths and induced abortions after 12 completed gestational weeks in the birth registry. These data included 1227 pregnancies during the follow-up period in addition to the live births being studied. Of these 1227 pregnancies ending in a fetal death or induced abortion, 64 (rate 522 per 10 000 pregnancies) had a major congenital anomaly registered. When we examined the risk of any major congenital anomaly according to infection including these pregnancies, the results were similar to the main results (adjusted odds ratio 0.98, 95% CI 0.86 to 1.11 including these additional pregnancies *v* 0.97, 0.85 to 1.11 in the main analysis) and with vaccination against covid-19 (1.07, 0.97 to 1.17 *v* 1.06, 0.97 to 1.17). No information on terminations with data on anomalies were available for Denmark and Sweden.

## Discussion

### Principal findings

Our Nordic registry based study did not find an increased risk of any major congenital anomalies among infants whose mothers had covid-19 infection or covid-19 vaccination during the first trimester. No notable heterogeneity in the risk was apparent according to viral variants, although larger studies are needed to provide more robust evidence.

### Strengths and limitations

Strengths of the current study include the large population based sample, the inclusion of data from several countries, and the evaluation of subgroups of anomalies. The rate of major congenital anomalies in our study is higher than that reported in the EUROCAT statistics for the included countries. This difference is probably explained by the lack of follow-up after birth for most regions in the EUROCAT statistics, changes in the definitions according to the latest updated version of the EUROCAT guidelines, and misclassification resulting in false positive registrations^32^—for example, when a child is under evaluation or being diagnosed. We do not expect any of these points to differ according to the exposures of interest, and any such non-differential misclassification could therefore have resulted in an underestimation of the associations of interest. As an example, we examined the limb anomaly subgroup further because the Danish rate for limb anomalies was especially high—primarily because of inclusion of hip anomalies recorded between birth and six weeks of age, which have been shown to have a high false positive rate.^33^ When excluding these diagnoses, the rate more than halved, but the overall estimates for the risk of limb anomalies according to covid-19 infection or vaccination were not appreciably affected.

The study also has limitations. Our analysis is restricted to live births. We chose not to include stillbirths in the main analysis because the presence of congenital anomalies is poorly recorded for this group, and we did not have information on fetal deaths or induced abortions for all countries. It is unlikely that our analyses of vaccination against covid-19 and risk of congenital anomalies is biased owing to exclusion of fetal deaths because no increased risk of miscarriage or stillbirth according to vaccination was observed.^11^ ^34^ However, the results for infection with covid-19 might be underestimated because an increased risk of stillbirth with covid-19 infection has been reported previously in our study population, and we do not know if congenital anomalies were contributing factors in these stillbirths.^30^ Differences in the testing strategy could have influenced our ability to identify women infected with covid-19.

We also acknowledge that we did not capture self-administered antigen tests for covid-19. However, for most of the study period, women who tested positive using an antigen test were advised to get a confirmatory PCR test. In Denmark, from March 2021 until March 2022, 80-90% of positive antigen tests were followed up by a confirmatory PCR test.^35^ Similar estimates are unfortunately not available for Sweden and Norway. It is possible that women with a higher risk of having a baby with a congenital anomaly had a greater likelihood of getting tested, for example older women and those with various underlying chronic conditions and using drugs, or women with a previous child with anomalies. It is also possible that pregnancies followed for congenital anomalies in specialised antenatal care or fetal medicine units were more likely to be tested for covid-19. We only had three digit ICD-10 codes available in Norway and Sweden, and we chose to be conservative in the exclusion of minor anomalies, which might have resulted in an underestimation of some of the associations of interest.

There might also be unmeasured or residual confounding influencing our results, for example through unmeasured factors such as differences in underlying genetic risk, use of drugs, or from measurement error or categorisation of included confounders. However, because none of the included factors are very strong risk factors for congenital anomalies, we do not expect the categorisation to be a large problem. The adjustment for underlying chronic conditions should account partially for use of drugs for these conditions. Moreover, we believe it is the underlying conditions, and not the drugs themselves, which might affect the likelihood of covid-19 infection and vaccination. To explore the scope of drug use, we checked how many pregnant women in Sweden and Norway were taking teratogens from a predefined list,^36^ and found that only 104 pregnancies across the two countries had maternal exposure during the first trimester. Because the use of these drugs was so rare, it is unlikely that it influenced our results. The role of underlying genetic risk might be further evaluated in larger datasets using a sibling comparison. Unmeasured confounding could have led us to overestimate the associations of interest. However, because our findings are largely null, if associations were even weaker then our conclusion of no indication of adverse effects of vaccinations still holds.

### Comparison with other studies

Our findings are in line with previous studies indicating no strong evidence for an increased risk of any major congenital anomalies after infection with^13^ ^14^ or vaccination against^15^ ^16^ ^17^ ^18^ ^19^ covid-19. Most of these existing studies had a modest sample size and inadequate power,^13^ ^15^ ^17^ ^19^ and were therefore not able to examine subgroups of congenital anomalies.^15^ ^16^ ^17^ ^19^ The studies that examined subgroups of anomalies indicated no notable increased risk after infection or vaccination.^14^ ^18^ These studies also had limited postnatal follow-up, and are likely to have underestimated the number of anomalies. Therefore, we add to the current evidence with our results showing that there appears to be no robust evidence of an increased risk of any of the subgroups of congenital anomalies as defined by the EUROCAT classification. Additionally, we provide some exploratory evidence that there do not appear to be any major differences in the risk according to covid-19 viral variants, although these results should be further explored in future studies.

### Policy implications

Evidence supports an increased risk of certain pregnancy complications, including preterm birth and stillbirth, among women with covid-19 infection during pregnancy.^3^ ^30^ We did not find any evidence of an increased risk of congenital anomalies after covid-19 infection, but newer variants were not included. However, current knowledge is in line with the new viral variants becoming less harmful.^37^ Vaccination of pregnant women protects the women and the infants from adverse outcomes. Furthermore, we did not find any indication that vaccination against covid-19 during the first trimester increased the risk of anomalies, providing additional evidence about the safety of vaccination in pregnant women. Overall, our findings support the current recommendations to vaccinate pregnant women against covid-19.

### Conclusions

Covid-19 infection and vaccination during the first trimester of pregnancy were not associated with risk of congenital anomalies.

**What is already known on this topic**

Existing studies on the risk of major congenital anomalies after infection with or vaccination against covid-19 are limited
Because the first trimester is the most important time for covid-19 exposure, and postnatal follow-up is necessary to identify anomalies not observed at birth, studies of any increased risk of major congenital anomalies have only recently become possible

**What this study adds**

Covid-19 infection or vaccination during the first trimester was not associated with congenital anomalies
Any differences in the risk of congenital anomalies according to viral variants of covid-19 should be further examined in future studies

## Data Availability

The data used in this study are available through application to Pregnancy Register (Datauttag | Graviditetsregistret, https://www.sfog.se/start/kvalitet/kvalitetsregister/graviditetsregistret/), National Board of Health and Welfare (https://bestalladata.socialstyrelsen.se/data-for-forskning/) and Statistics Sweden (https://www.scb.se/vara-tjanster/bestall-data-och-statistik/) in Sweden; Statistics Denmark (https://www.dst.dk/en/TilSalg/Forskningsservice/Dataadgang), the Danish Health Data Authorities (https://sundhedsdatastyrelsen.dk/da/forskerservice/om-forskerservice/nyheder_forskerservice/vaccinedata_031023), and Statens Serum Institute (https://miba.ssi.dk/forskningsbetjening) in Denmark; and the Norwegian Institute of Public Health in Norway (helsedata.no/en/). The analysis scripts are available upon request to the corresponding author.

## Web extra

Extra material supplied by authors

Web appendix: Supplementary appendix

## Notes

Contributors: MCM, JS, and SKU conceived and designed the study. OS, AMNA, and MCM obtained access to data. MCM, JS, and SKU conducted the data analysis. MCM drafted the initial version of the manuscript. AKO and SEH provided important insight during the data analysis. All authors contributed in the interpretation of the data and critically revised the manuscript. All authors had full access to the data in the study and can take responsibility for the integrity of the data and the accuracy of the data analysis. MCM is the guarantor. The corresponding author attests that all listed authors meet authorship criteria and that no others meeting the criteria have been omitted.

Transparency: The lead author (the manuscript’s guarantor) affirms that the manuscript is an honest, accurate, and transparent account of the study being reported; that no important aspects of the study have been omitted; and that any discrepancies from the study as planned (and, if relevant, registered) have been explained.

Dissemination to participants and related patient and public communities: Dissemination to the general population will be made through media outreach, including a press release and communication through social media outlets by the host institution, on publication of this study.

Provenance and peer review: Not commissioned; externally peer reviewed.
