# Supplementary material for: Covid-19 infection and vaccination during first trimester and risk of congenital anomalies: Nordic registry based study
Source: BMJ. 2024 Jul 17;386:e079364. doi: 10.1136/bmj-2024-079364 (PMC12036550; doi:10.1136/bmj-2024-079364)
Supplement: Supplementary file 1 — Web appendix: Supplementary appendix [file magm079364.ww.pdf]

Supplementary Appendix

This supplementary material has been provided by the authors to give readers additional information about their work.

Supplement to: Covid-19 infection and vaccination during first trimester and risk of congenital anomalies

Maria C. Magnus, Jonas Söderling, Anne K Örtqvist, Anne-Marie Nybo Andersen. Olof Stephansson, Siri E. Håberg, Stine Kjaer Urhoj

Table of contents

Data sources and linkages in Sweden……………………………………………………………………………………………p. 3
Data sources and linkages in Norway……………………………………………………………………………………………p. 4
Data sources and linkages in Denmark…………………………………………………………………………………………p. 5
eFigure 1 Distribution of calendar day for last menstrual period, infection with Covid-19 and vaccination against Covid-19……………………………………………………………………………………………………p.6
eTable 1 Recommendations for vaccination of pregnant women against Covid-19………………………p. 7
eTable 2 International Classification of Diseases version 10 (ICD-10) codes used to define major congenital anomalies according to the EUROCAT categories……………………………………………………p. 8
eTable 3 International Classification of Diseases (ICD) version 10 codes used to define pre-existing chronic conditions………p. 10
eTable 4 Maternal background characteristics according to infection with Covid-19 during first trimester stratified by each country………………………………………………………………………………………p. 11
eTable 5 Risk of congenital anomalies according to exposure to Covid-19 variants during the first trimester……p. 13
eTable 6 Risk of congenital anomalies according to exposure to infection with Covid-19 during the first trimester restricting to those with at least 12 months of follow-up……………………………………p. 14
eTable 7 Risk of congenital anomalies according to exposure to infection with Covid-19 during the first trimester excluding children with genetic malformations…………………………………………………p. 15
eTable 8 Maternal background characteristics according to vaccination against Covid-19 during first trimester stratified by each country………………………………………………………………………………………p. 16
eTable 9 Risk of congenital anomalies according to exposure to vaccination against Covid-19 during the first trimester restricting to those with at least 12 months of follow-up…………………………………p. 18
eTable 10 Risk of congenital anomalies according to exposure to vaccination against Covid-19 during the first trimester excluding children with genetic malformations………………………………………………p. 19
eTable 11 Risk of congenital anomalies according to exposure to vaccination against Covid-19 during the first trimester excluding those who remained unvaccinated at the end of follow-up……………p. 20
eTable 12 Risk of congenital anomalies according to exposure to vaccination against Covid-19 during the first trimester by vaccine type……………………………………………………………………………………p. 21

Data sources and linkages in Sweden

The Swedish Pregnancy register (SPR)

Data in this study was provided through the Swedish Pregnancy Register. This quality register was initiated in 2013, and includes 94% of all births in Sweden (18 of 21 regions). Demographical, reproductive and maternal health care data, starting at the first visit to the antenatal care clinic around the ninth gestational week, are transferred from electronic medical records within 24 hours from a reported birth. The register includes information on birth outcomes in addition to maternal background characteristics, health during pregnancy, pregnancy and neonatal outcomes.

The Swedish Neonatal Quality Register (SNQ)

Th SNQ includes all infants born alive in Sweden who were admitted for neonatal care within 27 days after birth. For this study we used information on neonatal care admission.

The national vaccination register in Sweden

As of 1 January 2013, healthcare providers must report all vaccinations administered within the Swedish vaccination programs to the Swedish vaccination register, held by the Public Health Agency of Sweden. The register includes type and date of all Covid-19 vaccinations.

Swedish Register for Communicable Diseases (SmiNet)

SmiNet is the Swedish Public Health Agency's national register for communicable diseases. On February 1, 2020, SARS-CoV-2 was included in the Swedish Communicable Diseases Act, making it mandatory to report all laboratory to confirmed Polymerase Chain Reaction (PCR) cases within 24 hours to the register. From this register we used information on positive tests as well as the date of a positive PCR test.

Data sources and linkages in Norway

The Emergency Preparedness Register for Covid-19
Data in this study were provided through the Emergency preparedness register for Covid-19 (Beredt C19) administered by the Norwegian Institute of Public Health, according to the Health Preparedness Act §2 to 4. This registry was established in 2020 to provide authorities with up to date information on prevalence, causal relationships, and consequences of the Covid19 epidemic in Norway. Beredt C19 includes information already collected in the healthcare service, national health registries and administrative registers with information about the Norwegian population. The data subjects' right is safeguarded as they can contact the data controller for all different sources included in Beredt C19 in the usual way. Through Beredt C19 we used data from the following sources:

The Norwegian Immunisation Register (SYSVAK)
SYSVAK is a register of vaccines in the Norwegian vaccination program, with mandatory registrations of all Covid-19 vaccinations (dates and type).

Norwegian Surveillance System for Communicable Diseases (MSIS)
There is mandatory reporting of selected infectious diseases to this National Health register. Reporting of all Covid-19 tests is mandatory, and this register contains date of testing and test results.

Statistics Norway (SSB)
Administrative data is mandatorily reported to Statistics Norway. We used information from this database on household income in 2018, type of education and years of education completed by 2019.

The Medical Birth Registry of Norway (MBRN)
The Norwegian national birth registry includes information on all pregnancies ending in gestational week 12 or later. The registry includes information on birth outcomes in addition to maternal background characteristics, health during pregnancy, pregnancy outcomes and neonatal health.

Data sources and linkages for Denmark

The Danish National Patient Register (DNPR)

The Danish National Patient Registry was established in 1977. The register contains individual to level longitudinal registration of all contacts to Danish hospitals (inpatient admissions and non to primary outpatient admissions), including dates and codes for diagnosis, births, medical surgery etc. Diagnosis codes are coded according to the International Classification of Diseases system. The validity and the completeness of the register vary according to clinical specialties; for the clinical specialty of obstetrics and gynecology the validity is generally considered to be high. In this study, the DNPR was used to identify all women giving birth after 22 gestational weeks, as the Danish Medical Birth Register has not been updated and released to Statistics Denmark since 2018 (as per early November 2023).

Danish Microbiology Database (MiBa)

The Danish Microbiology Database (MiBa) was established in 2010 and contains complete data on all microbiological samples performed in Denmark from general practices, test centres and hospitals. Besides SARS-CoV-2 test results from PCR tests, MiBa also includes rapid antigen tests from December 2020 performed at official test centres and other places reporting the test results to the database. Both SARS-CoV-2 PCR test and rapid antigen tests are free of charge in Denmark (not including home test kits), easily accessible and has been widely used. In case of a positive rapid antigen test result, the person was recommended to have a PCR test to confirm the result. The testing strategies varied during the study period. The testing rapidly intensified during the first months of the pandemic and included intense contact tracing.

Vaccination Register

The Danish Vaccination Register (DDV) covers all vaccines administered to Danish patients and the reporting to the register has been mandatory since 2015 (e.g. all Covid-19 vaccinations are registered with date and type). Covid-19 vaccination was recommended for pregnant women in their second and third trimester from July 21, 2021.

Statistics Denmark

Information about educational levels, income, living with a partner and region of birth was obtained from population registers at Statistics Denmark.

eFigure 1 Distribution of calendar day for last menstrual period, infection with Covid-19 and vaccination against Covid-19

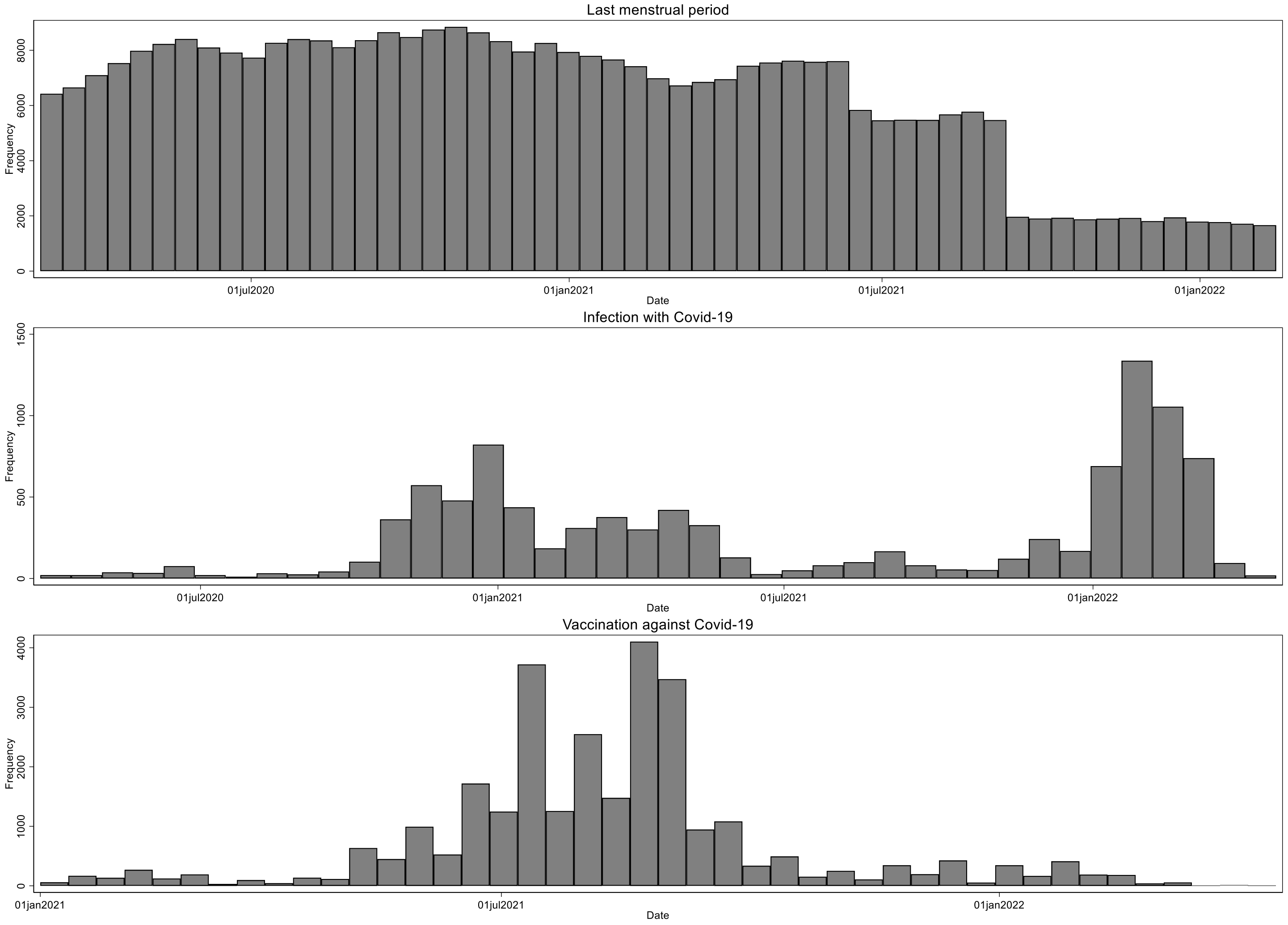

eTable 1 Recommendations for vaccination of pregnant women against Covid-19

Recommendation	Sweden	Denmark	Norway
All pregnant women at high risk (due to underlying chronic conditions or occupation) should be vaccinated from second trimester onwards	April 2021		April 2021<br>(Vaccination of pregnant women can be considered if the benefits are believed to outweigh the risks, also for pregnant women with high risk of being infected who do not have underlying diseases)
All pregnant women should be vaccinated from second trimester onwards	May 2021<br>(Pregnant women with risk factors for severe disease have been able to be vaccinated or revaccinated in the first trimester, following an individual assessment by a doctor)	July 2021<br>(Pregnant women with risk factors for severe disease have been able to be vaccinated or revaccinated in the first trimester, following an individual assessment by a doctor)	August 2021<br>(All pregnant women are recommended to get vaccinated from 2 trimester onwards. Vaccination during first trimester can be considered due to underlying diseases or particularly high risk of being infected)
All pregnant women should be vaccinated regardless of trimester	Never.<br>In 2nd or 3rd trimester since May 2021 (only in 1st trimester after individual assessment by doctor).	Never.<br>In 2nd or 3rd trimester since July 2021 (only in 1st trimester if risk factors for severe disease AND individual assessment by doctor).<br>In 1st trimester since September 2023 (if not vaccinated or if risk factors for severe disease)	January 2022<br>(All pregnant women are recommended to get vaccinated or receive a booster dose regardless of trimester)
All pregnant women recommended a booster if more than 20 weeks had passed since the last vaccine dose was administered	August 2023 (24 weeks since last administered dose)		November 2022

Throughout the pandemic, pregnant women were recommended seasonal influenza vaccination. Co-administration was conducted from Fall 2023 in Sweden and Norway, although not in Denmark.sssss

eTable 2 International Classification of Diseases version 10 (ICD-10) codes used to define major congenital anomalies according to the EUROCAT categories

EUROCAT categories	ICD-10 codes	Excluded minor anomalies[*]
Any	Q-chapter, D215, D821, D1810 (=D1814A in DK)	Q046, Q078 (Q078D and Q078G in DK), Q078, Q095, Q101, Q102, Q103, Q105, Q135, Q170, Q171, Q172, Q173, Q174, Q175, Q179, Q180, Q181, Q182, Q184, Q185, Q186, Q187, Q188, Q189, Q211 (Q211C in DK), Q246, Q250 if GA<37 weeks, Q254 (Q254E in DK), Q256 if GA<37 weeks, Q261, Q270, Q314, Q314 (Q318H in DK), Q315, Q320, Q322, Q330, Q331, Q331, Q357, Q381, Q382, Q385 (Q385B in DK), Q400, Q401, Q402, Q430, Q432, Q438, Q438, Q444, Q458 (Q458B in DK), Q501, Q501, Q501, Q502, Q505, Q523, Q525, Q527, Q530, Q544, Q552 (Q552F and Q552B in DK), Q610 (Q610A in DK), Q627, Q633, Q653-Q656, Q658, Q659, Q661, Q662, Q663, Q664, Q665, Q666, Q667, Q668, Q668, Q669, Q670, Q671, Q672, Q673, Q674 (Q674B and Q674C in DK), Q675, Q676, Q677, Q678, Q680 (Q680A in DK), Q681, Q682 (Q682A in DK), Q683, Q684, Q685, Q740 (Q740G in DK), Q752, Q753, Q760, Q764 (Q764L in DK), Q765, Q766, Q767 (Q767C in DK), Q825 (Q825E and Q825Q in DK), Q828, Q833, Q845, Q846, Q891, Q899, Q952
Congenital heart defects	Q20-Q26	Q211 (Q211C in DK), Q246, Q250 if GA <37 weeks, Q254 (Q254E in DK), Q256 if GA<37 weeks, Q261
Nervous system	Q00, Q01, Q02, Q03, Q04, Q05, Q06, Q07, Q870 (Q870D in DK)	Q046, Q078
Eye	Q10-Q15	Q101-Q103, Q105, Q135
Ear, Face and Neck	Q16, Q17, Q18	Q170-Q175, Q179, Q180-Q182, Q184-Q187, Q188, Q189
Respiratory	Q300, Q32-Q34	Q320, Q322, Q330, Q331, Q336
Oro-facial clefts	Q35-Q37	Q357. Exclude, not minor: if Q35-Q37 **AND** Q041 or Q042 or Q870 (Q870D in DK)
Gastro-intestinal	Q38-Q45, Q790	Q381, Q382, Q385 (Q385B in DK), Q400, Q401, Q402, Q430, Q432, Q438, Q444, Q458 (Q458B in DK) Exclude, not minor: if Q411-Q418 **AND** Q793 or Q792; if Q433 **AND** Q793 or Q792
Abdominal wall defects	Q792, Q793, Q795	
Kidney and urinary (CAKUT)	Q60-Q64, Q794	Q610 (Q610A in DK), Q627, Q633<br>Exclude, not minor: If Q620 or Q621 or Q623 **AND** Q627 or Q00 or Q01 or Q05
Genital	Q50-Q52, Q54- Q56	Q523, Q525, Q527, Q552 (Q552F and Q552B in DK), Q501, Q502, Q505, Q544
Limb	Q65-Q74	Q653-Q656, Q662-Q669, Q670-Q678, Q680 (Q680A in DK), Q681, Q682 (Q682A in DK), Q683-Q685, Q740 (Q740G in DK), Q658, Q659, Q661

		Exclude, not minor: If Q660 **AND** Q00 or Q01 or Q05 or Q601 or Q606; If Q650-Q652 **AND** Q00 or Q01 or Q05

[*]In Sweden and Norway, there is only information on ICD-10 codes with the following format QXXX. We were therefore conservative in our exclusion of minor anomalies based on this level of information. More detailed codes were available for the majority of subgroups in Denmark (QXXXX).

eTable 3 International Classification of Diseases (ICD) version 10 codes used to define pre-existing chronic conditions

Chronic conditions	ICD-10 codes
Asthma	J45, J46
Diabetes mellitus	E10, E11, E12, E13, E14
Epilepsy	G40, G41
Chronic hypertension	I10, I11, I12, I13, I14, I15
Chronic kidney disease	N10-19
Thrombosis	I21, I22, I26, I63, I8I, I82
Cardiovascular disease	I2, I4, I5, I6, I7

eTable 4 Maternal background characteristics according to infection with Covid-19 during first trimester stratified by each country

Maternal background characteristics, n(%)	Sweden (n=161,284)		Denmark (n=78,035)		Norway (n=103,747)	
	Not infected during first trimester (n=157,240)	Infected during first trimester (n=4,044)	Not infected during first trimester (n=76,856)	Infected during first trimester (n=1,179)	Not infected during first trimester (n=98,741)	Infected during first trimester (n=5,006)
**Age at start of pregnancy**						
<25 years	16,862 (10.7)	430 (10.6)	7,812 (10.2)	129 (10.9)	7,543 (7.6)	320 (4.1)
25-29 years	51,677 (32.9)	1,405 (34.7)	28,354 (36.9)	476 (40.4)	29,705 (30.1)	1,412 (28.2)
30-34 years	58,450 (37.2)	1,465 (36.2)	27,400 (35.7)	387 (32.8)	39,308 (39.8)	2,049 (40.9)
35-39 years	25,151 (16.0)	598 (14.8)	11,043 (14.4)	164 (13.9)	18,226 (18.5)	1,001 (20.0)
40 years or older	5,100 (3.2)	146 (3.6)	2,247 (2.9)	23 (2.0)	3,959 (4.0)	224 (4.5)
**Parity**						
0	66,986 (42.6)	1,655 (40.9)	36,109 (47.0)	532 (45.1)	42,470 (43.0)	1,891 (37.8)
1	58,941 (37.5)	1,490 (36.8)	29,122 (37.9)	441 (37.4)	36,789 (37.3)	1,920 (38.4)
2	21,456 (13.6)	638 (15.8)	9,196 (12.0)	155 (13.2)	14,113 (14.3)	839 (16.8)
3 or higher	9,857 (6.3)	261 (6.5)	2,429 (3.2)	51 (4.3)	5,369 (5.4)	356 (7.1)
**Highest obtained educational level**						
9 years or less	9,384 (6.0)	182 (4.5)	7,726 (10.1)	151 (12.8)	13,086 (13.3)	707 (14.1)
10-12 years	49,113 (31.2)	1,411 (34.9)	20,358 (26.5)	303 (25.7)	18,791 (19.0)	995 (19.9)
More than 12 years	97,753 (62.2)	2,441 (60.4)	48,362 (62.9)	717 (60.8)	56,014 (56.7)	2,737 (54.7)
Unknown	990 (0.6)	10 (0.2)	410 (0.5)	8 (0.7)	10,850 (11.0)	567 (11.3)
**Income category in tertile**						
1st tertile	51,316 (32.6)	1,216 (30.1)	20,400 (26.5)	379 (32.2)	30,562 (31.0)	1,734 (34.6)
2nd tertile	51,044 (32.5)	1,489 (36.8)	23,936 (31.1)	358 (30.4)	30,724 (31.1)	1,539 (30.7)
3rd tertile	51,221 (32.6)	1,312 (32.4)	25,147 (32.7)	331 (28.1)	30,658 (31.1)	1,425 (28.5)
Unknown	3,659 (2.3)	27 (0.7)	7,373 (9.6)	111 (9.4)	6,797 (6.9)	308 (6.2)
**Country of birth/origin**†						
Scandinavia	112,148 (71.3)	2,913 (72.0)	64,370 (83.8)	938 (79.6)	73,259 (74.4)	3,580 (71.6)
Other European countries	12,154 (7.7)	331 (8.2)	5,828 (7.6)	79 (6.7)	10,860 (11.0)	623 (12.5)
Middle East/Africa	25,453 (16.2)	660 (16.3)	3,839 (5.0)	123 (10.4)	6,866 (7.0)	467 (9.3)

Other/unknown	7,485 (4.8)	140 (3.5)	2,819 (3.7)	39 (3.3)	7446 (7.6)	330 (6.6)
**Any chronic disease[b]**						
No	136,392 (86.7)	3,472 (85.9)	74,122 (96.4)	1,144 (97.0)	89,161 (90.3)	4,520 (90.3)
Yes	20,848 (13.3)	572 (14.1)	2,734 (3.6)	35 (3.0)	9,580 (9.7)	486 (9.7)
**Smoking during pregnancy**						
No	146,609 (93.2)	3,786 (93.6)	68,639 (89.3)	1,063 (90.2)	85,003 (86.1)	4,208 (84.1)
Yes	4,929 (3.1)	95 (2.3)	5,249 (6.8)	65 (5.5)	1,666 (1.7)	68 (1.4)
Unknown	5,702 (3.6)	163 (4.0)	2,968 (3.9)	51 (4.3)	12,072 (12.2)	730 (14.6)
**Pre-pregnancy body-mass index**						
<18.5	3,295 (2.1)	56 (1.4)	2,623 (3.4)	38 (3.2)	3,054 (3.1)	142 (2.8)
18.5-24.9	79,555 (50.6)	1,990 (49.2)	40,921 (53.2)	591 (50.1)	53,591 (54.3)	2,716 (54.3)
25.0-29.9	42,700 (27.2)	1,142 (28.2)	19,559 (25.5)	334 (28.3)	22,471 (22.8)	1,181 (23.6)
30 or higher	25,835 (16.4)	726 (18.0)	12,350 (16.1)	192 (16.3)	13,897 (14.1)	736 (14.7)
Unknown	5,855 (3.7)	130 (3.2)	1,403 (1.8)	24 (2.0)	5,728 (5.8)	231 (4.6)

[*] The other category includes North America, South America, Latin America, Asia, Australia, and New Zealand.

[†] Includes hypertension, chronic kidney disease, cardiovascular disease, asthma, thrombosis, diabetes mellitus (type 1 and 2), and epilepsy.

eTable 5 Risk of congenital anomalies according to exposure to Covid-19 variants during the first trimester

EUROCAT categories of major congenital anomalies*	Index variant		Alpha variant		Delta variant	
	OR adjusted for estimated start of pregnancy (95% CI)	Fully adjusted OR (95% CI) †	OR adjusted for estimated start of pregnancy (95% CI)	Fully adjusted OR (95% CI) †	OR adjusted for estimated start of pregnancy (95% CI)	Fully adjusted OR (95% CI) †
Any	0.92 (0.78 to 1.09)	0.93 (0.78 to 1.19)	0.95 (0.77 to 1.18)	0.96 (0.76 to 1.20)	0.96 (0.84 to 1.11)	0.98 (0.86 to 1.13)
Congenital heart defects	1.10 (0.82 to 1.48)	1.08 (0.80, 1.46)	0.88 (0.37 to 2.08)	0.86 (0.34 to 2.16)	1.04 (0.82 to 1.31)	1.04 (0.83 to 1.31)
Nervous system	1.14 (0.36 to 3.59)	1.10 (0.34 to 3.52)	NA	NA	0.48 (0.15 to 1.55)	0.57 (0.17 to 1.89)
Eye	1.15 (0.51 to 2.59)	1.10 (0.48 to 2.49)	0.59 (0.15 to 2.35)	0.63 (0.15 to 2.59)	0.92 (0.44 to 1.91)	0.87 (0.42 to 1.81)
Oro-facial clefts	1.17 (0.37 to 3.71)	1.17 (0.36 to 3.81)	1.88 (0.70 to 5.05)	2.21 (0.80 to 6.09)	1.14 (0.56 to 2.29)	0.18 (0.58 to 2.40)
Gastro-intestinal	1.01 (0.30 to 3.40)	1.06 (0.36 to 3.15)	1.01 (0.42 to 2.44)	0.99 (0.40 to 2.43)	1.10 (0.61 to 1.97)	1.07 (0.59 to 1.91)
Kidney and urinary (CAKUT)	1.00 (0.58 to 1.72)	1.04 (0.60 to 1.80)	1.41 (0.72 to 2.77)	1.40 (0.65 to 3.02)	0.82 (0.52 to 1.28)	0.84 (0.53 to 1.31)
Genital	0.88 (0.49 to 1.58)	0.87 (0.48 to 1.57)	0.78 (0.35 to 1.75)	0.78 (0.35 to 1.76)	1.06 (0.69 to 1.61)	1.09 (0.72 to 1.66)
Limb	0.95 (0.62 to 1.46)	0.93 (0.55 to 1.57)	0.70 (0.42 to 1.19)	0.72 (0.42 1.21)	1.11 (0.84 to 1.47)	1.14 (0.86 to 1.51)

*Ear, face and neck anomalies, respiratory anomalies and abdominal wall defects not evaluated because of fewer than 5 exposed cases across the three countries.

†Adjusted for maternal age, parity, highest obtained educational level, household income level, smoking during pregnancy, pre-pregnancy body-mass index, country of birth/origin, estimated start of pregnancy (last menstrual period), chronic diseases, and vaccination against Covid-19 in first trimester.

eTable 6 Risk of congenital anomalies according to exposure to infection with Covid-19 during the first trimester restricting to those with at least 12 months of follow-up

EUROCAT categories of major congenital anomalies*	Unexposed (n=283,019) Number cases	Exposed (n=6,166) Number cases	OR adjusted for estimated start of pregnancy (95% CI)	Fully adjusted OR (95% CI) †
Any	14707	284	0.94 (0.83 to 1.06)	0.95 (0.84 to 1.07)
Congenital heart defects	4005	99	1.19 (0.97 to 1.46)	1.19 (0.97 to 1.46)
Eye	503	9	0.81 (0.42 to 1.57)	0.81 (0.41 to 1.57)
Oro-facial clefts	409	9	1.11 (0.57 to 2.16)	1.12 (0.58 to 2.19)
Gastro-intestinal	1012	17	0.87 (0.50 to 1.53)	0.90 (0.53 to 1.55)
Kidney and urinary (CAKUT)	1356	24	0.85 (0.44 to 1.68)	0.87 (0.44 to 1.73)
Genital	1246	21	0.80 (0.52 to 1.23)	0.80 (0.52 to 1.24)
Limb	3269	57	0.94 (0.65 to 1.35)	0.96 (0.63 to 1.45)

*Nervous system defects, ear, face and neck anomalies, respiratory anomalies and abdominal wall defects not evaluated because of fewer than 5 exposed cases across the three countries.

† Adjusted for maternal age, parity, highest obtained educational level, household income level, smoking during pregnancy, pre-pregnancy body-mass index, country of birth/origin, estimated start of pregnancy (last menstrual period), chronic diseases, and vaccination against Covid-19 in first trimester.

eTable 7 Risk of congenital anomalies according to exposure to infection with Covid-19 during the first trimester excluding children with genetic malformations

EUROCAT categories of major congenital anomalies*	Unexposed (n=331,769) Number cases	Exposed (n=10,196) Number cases	OR adjusted for estimated start of pregnancy (95% CI)	Fully adjusted OR (95% CI) †
Any	16148	461	0.94 (0.86 to 1.04)	0.96 (0.87 to 1.05)
Congenital heart defects	4363	152	1.09 (0.88 to 1.35)	1.10 (0.90 to 1.35)
Nervous system	386	6	0.62 (0.28 to 1.41)	0.66 (0.29 to 1.52)
Eye	546	16	0.94 (0.57 to 1.56)	0.90 (0.54 to 1.50)
Oro-facial clefts	416	16	1.16 (0.70 to 1.94)	1.18 (0.71 to 1.96)
Gastro-intestinal	1079	23	0.82 (0.48 to 1.41)	0.86 (0.53 to 1.41)
Kidney and urinary (CAKUT)	1508	43	0.98 (0.70 to 1.36)	1.00 (0.69 to 1.45)
Genital	1393	42	0.94 (0.69 to 1.29)	0.96 (0.70 to 1.32)
Limb	3780	100	0.93 (0.70 to 1.23)	0.93 (0.67 to 1.29)

*Ear, face and neck anomalies, respiratory anomalies and abdominal defects not evaluated because of fewer than 5 exposed cases across the three countries.

†Adjusted for maternal age, parity, highest obtained educational level, household income level, smoking during pregnancy, pre-pregnancy body-mass index, country of birth/origin, estimated start of pregnancy (last menstrual period), chronic diseases, and vaccination against Covid-19 in first trimester.

eTable 8 Maternal background characteristics according to vaccination against Covid-19 during first trimester stratified by each country

**Maternal background characteristics n(%)**	**Sweden (n=69,082)**		**Denmark (n=26,261)**		**Norway (n=56,918)**	
	Not vaccinated during first trimester (n=51,264)	Vaccinated during first trimester (n=17,818)	Not vaccinated during first trimester (n=24,016)	Vaccinated during first trimester (n=2,245)	Not vaccinated during first trimester (n=47,846)	Vaccinated during first trimester (n=9,072)
**Age at start of pregnancy**						
<25 years	6,027 (11.8)	1,229 (6.9)	2,427 (10.1)	197 (8.8)	3,639 (7.6)	646 (7.1)
25-29 years	16,893 (33.0)	5,397 (30.3)	8,843 (36.8)	727 (32.4)	14,293 (29.9)	2,677 (29.5)
30-34 years	18,498 (36.1)	7,296 (40.9)	8,526 (35.5)	790 (35.2)	18,850 (39.4)	3,661 (40.4)
35-39 years	8,040 (15.7)	3,258 (18.3)	3,478 (14.5)	434 (19.3)	9,060 (18.9)	1,736 (19.1)
40 years or older	1,806 (3.5)	638 (3.6)	742 (3.1)	97 (4.3)	2,004 (4.2)	352 (3.9)
**Parity**						
0	22,015 (42.9)	7,720 (43.3)	11,552 (48.1)	1,052 (46.9)	20,720 (43.3)	3,932 (43.3)
1	18,372 (35.8)	7,165 (40.2)	8,939 (37.2)	835 (37.2)	17,588 (36.8)	3,408 (37.6)
2	7,256 (14.2)	2,156 (12.1)	2,758 (11.5)	285 (12.7)	6,852 (14.3)	1,294 (14.3)
3 or higher	3,621 (7.1)	777 (4.4)	767 (3.2)	73 (3.3)	2,686 (5.6)	438 (4.8)
**Highest obtained educational level**						
9 years or less	3,750 (7.3)	424 (2.4)	2,519 (10.5)	145 (6.5)	6,623 (13.8)	1,081 (11.9)
10-12 years	17,372 (33.9)	4,379 (24.6)	6,469 (26.9)	434 (19.3)	9,198 (19.2)	1,787 (19.7)
More than 12 years	29,690 (57.9)	12,965 (72.8)	14,897 (62.0)	1,656 (73.8)	25,961 (54.3)	5,460 (60.2)
Unknown	452 (0.9)	50 (0.3)	131 (0.6)	10 (0.5)	6,064 (12.7)	744 (8.2)
**Income category in tertile**						
1st tertile	18,013 (35.1)	4,671 (26.2)	6,856 (28.6)	494 (22.0)	15,643 (32.7)	3,015 (33.2)
2nd tertile	15,998 (31.2)	5,892 (33.1)	7,589 (31.6)	757 (33.7)	14,354 (30.0)	2,768 (30.5)
3rd tertile	15,811 (30.8)	7,147 (40.1)	7,765 (32.3)	958 (42.7)	13,778 (28.8)	2,825 (31.1)
Unknown	1,442 (2.8)	108 (0.6)	1,806 (7.5)	36 (1.6)	4,071 (8.5)	464 (5.1)
**Country of birth/origin***						
Scandinavia	33,760 (65.9)	14,718 (82.6)	19,940 (83.0)	2,006 (89.4)	34,717 (72.9)	7,251 (79.9)
Other European countries	4,580 (8.9)	870 (4.9)	1,881 (7.8)	113 (5.0)	5,649 (11.9)	641 (7.1)
Middle East/Africa	10,392 (20.3)	1,360 (7.6)	1,302 (5.4)	48 (2.1)	3,681 (7.7)	485 (5.4)

Other/unknown	2,532 (4.9)	870 (4.9)	893 (3.7)	78 (3.5)	3604 (7.5)	694 (7.6)
**Any chronic disease**[†]						
No	44,671 (87.1)	15,258 (85.6)	23,189 (96.6)	2,136 (95.1)	43,244 (90.4)	8,026 (88.5)
Yes	6,593 (12.9)	2,560 (14.4)	827 (3.4)	109 (4.9)	4,602 (9.6)	1,046 (11.5)
**Smoking during pregnancy**						
No	47,571 (92.8)	16,830 (94.5)	21,520 (89.6)	2,074 (92.4)	40,692 (85.1)	7,759 (85.5)
Yes	1,738 (3.4)	306 (1.7)	1,632 (6.8)	104 (4.6)	725 (1.5)	141 (1.6)
Unknown	1,955 (3.8)	682 (3.8)	864 (3.6)	67 (3.0)	6,429 (13.4)	1,172 (12.9)
**Pre-pregnancy body-mass index**						
<18.5	1,131 (2.2)	293 (1.6)	807 (3.4)	52 (2.3)	1,463 (3.1)	267 (2.9)
18.5-24.9	25,289 (49.3)	9,072 (50.9)	12,645 (52.7)	1,215 (54.1)	25,917 (54.2)	4,836 (53.3)
25.0-29.9	14,372 (28.0)	4,678 (26.3)	6,207 (25.9)	598 (26.6)	11,108 (23.2)	2,084 (23.0)
30 or higher	8,582 (16.7)	3,100 (17.4)	4,010 (16.7)	349 (15.6)	6,855 (14.3)	1,439 (15.9)
Unknown	1,890 (3.7)	675 (3.8)	347 (1.4)	31 (1.4)	2,503 (5.2)	446 (4.9)

[*]The other category includes North America, South America, Latin America, Asia, Australia, and New Zealand.

[†] Includes hypertension, chronic kidney disease, cardiovascular disease, asthma, thrombosis, diabetes mellitus (type 1 and 2), and epilepsy.

eTable 9 Risk of congenital anomalies according to exposure to vaccination against Covid-19 during the first trimester restricting to those with at least 12 months of follow-up

EUROCAT categories of major congenital anomalies*	Unexposed (n=82,815) Number cases	Exposed (n=15,607) Number cases	OR adjusted for estimated start of pregnancy (95% CI)	Fully adjusted OR (95% CI) †
Any	4197	773	1.03 (0.95 to 1.11)	1.03 (0.95 to 1.12)
Congenital heart defects	1175	224	1.01 (0.87 to 1.18)	1.00 (0.86 to 1.17)
Nervous system	103	18	0.85 (0.29 to 2.54)	0.93 (0.35 to 2.46)
Eye	151	25	0.82 (0.51 to 1.33)	0.82 (0.51 to 1.33)
Respiratory	51	5	0.64 (0.24 to 1.69)	0.59 (0.22 to 1.59)
Oro-facial clefts	120	25	1.16 (0.74 to 1.84)	1.11 (0.70 to 1.76)
Gastro-intestinal	274	45	0.90 (0.54 to 1.49)	0.88 (0.58 to 1.35)
Abdominal wall defects	23	6	1.75 (0.52 to 5.94)	2.13 (0.77 to 5.94)
Kidney and urinary (CAKUT)	397	81	1.18 (0.92 to 1.51)	1.14 (0.89 to 1.47)
Genital	391	77	1.10 (0.79 to 1.53)	1.18 (0.91 to 1.53)
Limb	894	128	0.92 (0.75 to 1.12)	0.91 (0.75 to 1.11)

*Ear, face and neck anomalies not evaluated because there were fewer than 5 exposed cases across the three countries.

†Adjusted for maternal age, parity, highest obtained educational level, household income level, smoking during pregnancy, pre-pregnancy body-mass index, country of birth/origin, estimated start of pregnancy (last menstrual period), chronic diseases, and vaccination against Covid-19 in first trimester.

eTable 10 Risk of congenital anomalies according to exposure to vaccination against Covid-19 during the first trimester excluding children with genetic malformations

EUROCAT categories of major congenital anomalies	Unexposed (n=122,697) Number cases	Exposed (n=29,055) Number cases	OR adjusted for estimated start of pregnancy (95% CI)	Fully adjusted OR (95% CI) *
Any	5855	1314	1.03 (0.97 to 1.10)	1.04 (0.97 to 1.11)
Congenital heart defects	1610	389	1.05 (0.89 to 1.24)	1.06 (0.88 to 1.27)
Nervous system	125	28	0.85 (0.27 to 2.70)	0.92 (0.31 to 2.72)
Eye	209	50	0.92 (0.66 to 1.27)	0.93 (0.67 to 1.30)
Ear, face and neck	48	5	0.42 (0.16 to 1.08)	0.42 (0.16 to 1.08)
Respiratory	62	9	0.83 (0.41 to 1.69)	0.82 (0.40 to 1.69)
Oro-facial clefts	162	35	0.94 (0.65 to 1.38)	0.90 (0.61 to 1.33)
Gastro-intestinal	363	78	1.09 (0.70 to 1.69)	1.09 (0.69 to 1.72)
Abdominal wall defects	30	9	1.67 (0.75 to 3.70)	1.74 (0.77 to 3.95)
Kidney and urinary (CAKUT)	561	126	1.04 (0.81 to 1.34)	1.02 (0.77 to 1.34)
Genital	541	117	0.95 (0.78 to 1.17)	1.02 (0.83 to 1.26)
Limb	1366	255	1.02 (0.88 to 1.17)	0.99 (0.86 to 1.15)

*Adjusted for maternal age, parity, highest obtained educational level, household income level, smoking during pregnancy, pre-pregnancy body-mass index, country of birth/origin, estimated start of pregnancy (last menstrual period), chronic diseases, and vaccination against Covid-19 in first trimester.

eTable 11 Risk of congenital anomalies according to exposure to vaccination against Covid-19 during the first trimester excluding those who remained unvaccinated at the end of follow-up

EUROCAT categories of major congenital anomalies	Unexposed (n=91,038) Number cases	Exposed (n=29,135) Number cases	OR adjusted for estimated start of pregnancy (95% CI)	Fully adjusted OR (95% CI) *
Any	4680	1395	1.02 (0.96 to 1.09)	1.02 (0.95 to 1.09)
Congenital heart defects	1305	416	1.02 (0.90 to 1.14)	1.01 (0.90 to 1.14)
Nervous system	108	30	0.84 (0.32 to 2.24)	0.79 (0.28 to 2.22)
Eye	162	53	0.89 (0.64 to 1.25)	0.86 (0.61 to 1.21)
Ear, face and neck	45	6	0.41 (0.17 to 0.99)	0.41 (0.17 to 1.00)
Respiratory	59	9	0.60 (0.29 to 1.23)	0.55 (0.26 to 1.14)
Oro-facial clefts	138	46	1.03 (0.73 to 1.46)	1.05 (0.73 to 1.50)
Gastro-intestinal	287	84	1.04 (0.80 to 1.36)	1.02 (0.78 to 1.33)
Abdominal wall defects	16	9	2.06 (0.86 to 4.96)	2.38 (0.97 to 5.81)
Kidney and urinary (CAKUT)	450	134	1.04 (0.75 to 1.43)	1.03 (0.75 to 1.43)
Genital	393	120	1.01 (0.81 to 1.25)	1.02 (0.82 to 1.27)
Limb	1069	262	0.99 (0.86 to 1.15)	0.99 (0.86 to 1.14)

*Adjusted for maternal age, parity, highest obtained educational level, household income level, smoking during pregnancy, pre-pregnancy body-mass index, country of birth/origin, estimated start of pregnancy (last menstrual period), chronic diseases, and infection with Covid-19 in first trimester.

eTable 12 Risk of congenital anomalies according to exposure to vaccination against Covid-19 during the first trimester by vaccine type

EUROCAT categories of major congenital anomalies*	Unexposed/ reference (n=123126) Number cases	Pfizer-BioNTech (BNT162b2)			Moderna (mRNA to 1273)			Moderna versus Pfizer-BioNTech	
		Exposed (n=22322) Number cases	OR adjusted for estimated start of pregnancy (95% CI)	Fully adjusted OR (95% CI) †	Exposed (n=6813) Number cases	OR adjusted for estimated start of pregnancy (95% CI)	Fully adjusted OR (95% CI) †	OR adjusted for estimated start of pregnancy (95% CI)	Fully adjusted OR (95% CI) †
Any	6284	1060	1.02 (0.95 to 1.09)	1.03 (0.96 to 1.10)	335	1.02 (0.89 to 1.16)	1.02 (0.89 to 1.16)	0.98 (0.86 to 1.12)	0.98 (0.86 to 1.12)
Congenital heart defects	1745	318	1.02 (0.87 to 1.19)	1.05 (0.89 to 1.23)	98	0.99 (0.81 to 1.22)	1.01 (0.82 to 1.25)	0.99 (0.78 to 1.24)	0.99 (0.79 to 1.26)
Nervous system	147	22	0.99 (0.29 to 3.35)	1.04 (0.32 to 3.39)	8	1.12 (0.54 to 2.29)	1.13 (0.55 to 2.34)	0.92 (0.39 to 2.17)	0.94 (0.41 to 2.16)
Eye	227	48	1.02 (0.74 to 1.42)	1.02 (0.73 to 1.42)	5	0.45 (0.16 to 1.25)	0.45 (0.16 to 1.24)	0.38 (0.15 to 1.00)	0.42 (0.17 to 1.05)
Oro-facial clefts	179	34	1.10 (0.75 to 1.61)	1.06 (0.71 to 1.58)	12	1.25 (0.69 to 2.25)	1.20 (0.66 to 2.18)	1.13 (0.57 to 2.11)	1.24 (0.66 to 2.24)
Gastro-intestinal	397	63	0.92 (0.69 to 1.24)	0.99 (0.75 to 1.31)	21	0.80 (0.39 to 1.66)	1.03 (0.59 to 1.80)	1.09 (0.55 to 2.17)	1.15 (0.59 to 2.24)
Kidney and urinary (CAKUT)	593	100	1.06 (0.85 to 1.32)	1.04 (0.83 to 1.30)	34	1.08 (0.60 to 1.94)	1.03 (0.53 to 2.02)	1.11 (0.75 to 1.64)	1.08 (0.68 to 1.72)
Genital	559	88	0.90 (0.71 to 1.13)	0.96 (0.75 to 1.21)	32	1.06 (0.74 to 1.51)	1.10 (0.77 to 1.58)	1.17 (0.78 to 1.77)	1.13 (0.75 to 1.70)
Limb	1395	200	1.04 (0.89 to 1.21)	1.02 (0.87 to 1.20)	62	0.98 (0.67 to 1.43)	0.96 (0.65 to 1.42)	0.91 (0.59 to 1.39)	0.85 (0.61 to 1.18)

*Ear, face and neck anomalies, respiratory anomalies and abdominal wall defects not evaluated because there were fewer than 5 exposed cases across the three countries.

†Adjusted for maternal age, parity, highest obtained educational level, household income level, smoking during pregnancy, pre-pregnancy body-mass index, country of birth/origin, estimated start of pregnancy (last menstrual period), chronic diseases, and infection with Covid-19 in first trimester.
